# Perceptions of healthcare professionals and patients on the role of the pharmacist in TB management in Pakistan: A qualitative study

**DOI:** 10.3389/fphar.2022.965806

**Published:** 2022-12-15

**Authors:** Muhammad Atif, Kiran Munir, Iram Malik, Yaser Mohammed Al-Worafi, Irem Mushtaq, Nafees Ahmad

**Affiliations:** ^1^ Department of Pharmacy Practice, Faculty of Pharmacy, The Islamia University of Bahawalpur, Bahawalpur, Pakistan; ^2^ Department of Clinical Pharmacy, University of Science and Technology of Fujairah, Fujairah, United Arab Emirates; ^3^ Department of Education, Faculty of Education, The Islamia University of Bahawalpur, Bahawalpur, Pakistan; ^4^ Department of Pharmacy Practice, Faculty of Pharmacy and Health Sciences, University of Baluchistan, Quetta, Pakistan

**Keywords:** pharmacist, tuberculosis management, multidisciplinary approach, patient centered care, National Tuberculosis Control Program of Pakistan, Pakistan, FIP

## Abstract

**Background:** Globally, tuberculosis (TB) is the second major cause of death from infectious diseases, particularly in developing countries. A multidisciplinary approach to the management of TB may help to curb the disease burden.

**Objective:** The objective of this study was to outline the perceptions of healthcare professionals and patients regarding the potential role of pharmacists in TB management in Pakistan.

**Method:** This was a large-scale qualitative study conducted at the Chest Disease Unit (CDU) of the Bahawal Victoria Hospital (BVH), Punjab, Pakistan. Data were collected through semi-structured interviews with physicians, pharmacists, and patients recruited using a mix of convenient and snowball sampling. The sample size was decided through standard saturation point criteria. All interviews were audio recorded and transcribed verbatim. The data were analyzed to draw conclusions using a thematic analysis approach.

**Results:** Analysis of the data yielded 19 categories and seven themes. Physicians considered pharmacists qualified healthcare professionals, whereas patients considered them merely dispensers. Inventory management and dispensing of medicines were considered as major responsibilities of pharmacists. Physicians were extremely overburdened and wanted to delegate certain duties to pharmacists, subject to their prior extensive trainings. However, most of the physicians were unaware of the legal scope of pharmacy practice in Pakistan. With regard to the potential duties of pharmacists, physicians, pharmacists, and patients (patients—upon explaining the potential roles during the interview) endorsed monitoring, counseling, medicine brand selection, dose adjustment, inventory management, dispensing, and polypharmacy assessment as their potential roles. In view of all stakeholders, the rationale for integrating pharmacists in TB management included overburdened physicians, sub-standard patient care, medication safety issues, and patient dissatisfaction. The healthcare professionals highlighted that the major barriers to integrating pharmacists within the TB management system were limited interest of regulatory authorities and policy makers, followed by inadequate training and experience-driven questionable competency of pharmacists.

**Conclusion:** The study participants acknowledged the potential role of pharmacists in TB management. However, it was emphasized that healthcare policy makers should devise strategies to overcome the underlying barriers before assigning medicine-related clinical roles to pharmacists.

## Background

The global burden of tuberculosis (TB) is significant and will remain a major public health issue worldwide unless addressed with some vigor (World Health Organization, 2010). According to a recent report, two-thirds of the total disease burden is borne primarily by eight countries: India, China, Indonesia, the Philippines, Pakistan, Nigeria, Bangladesh, and South Africa (in descending order) (World Health Organization, 2017). Despite the fact that effective TB therapy has been available for more than half a century and the overall TB mortality rate has fallen by 9.2% (2015–2020), TB killed approximately 1.3 million people globally in 2020 (World Health Organization, 2017).

In the developing countries, insufficient access to diagnosis and treatment, co-existence of TB with other diseases such as diabetes and human immunodeficiency virus (HIV), and an increase in the incidence of multidrug-resistant TB (MDR-TB) are considerable challenges that need to be addressed for its effective management (Sia and Wieland, 2011; Atif et al., 2014). Moreover, the necessary education of TB patients about the disease and its treatment is important for the achievement of desirable outcomes (Kulkarni et al., 2013). In the traditional context, physicians prescribe medicines, and pharmacists compound and dispense these medicines (Richardson and Pollock, 2010; Gokcekus et al., 2012). However, there is evidence that a patient-centered approach based on the involvement of a multi-disciplinary team (including a pharmacist) is helpful for the effective management of a disease as calamitous as TB (Cipolle et al., 2012; Armor et al., 2014). Pharmacists can play a significant role in direct patient care. For example, counseling by pharmacists can minimize missing dose scenarios and aid in promoting adherence to anti-TB treatment, which are main barriers to favorable treatment outcomes in Pakistan (Atif et al., 2016a; Atif et al., 2018; Khan et al., 2019; Atif et al., 2020a; Atif et al., 2020b; Atif et al., 2021). Besides, appropriate medicine use and self-care practices can be improved through patient/caregiver education on medication use and disease control and prevention. Likewise, pharmacists can assist physicians in evidence based clinical practice by assessing patients for drug resistance and analyzing therapeutic problems and patients’ pharmaceutical care needs, such as pain control, nutrient replacement, rationale prescribing, and managing comorbidities. Similarly, pharmacists hold well-proven expertise in monitoring therapy for effectiveness, adverse drug reactions (ADRs) and drug interactions, posology, and post-discharge counseling, thereby ensuring medication safety (Mitrzyk, 2008; Mkele, 2010).

Pakistan ranks fifth among TB high burden countries (HBCs), which should be a point of concern for policymakers (National Tuberculosis Control Programme Pakistan, 2016; World Health Organization, 2021). This partially might be attributed to a shortage of TB management staff, lengthy treatment courses, side effects associated with anti-TB drugs, resistance to first-line anti-TB drugs, and the disappearance of signs and symptoms of TB after partial anti-tuberculosis treatment (ATT) (Atif et al., 2014; Atif et al., 2017a). Alongside poor health literacy, stigma associated with the disease, and poor financial status of TB patients and their families, these factors further complicate the TB treatment cascade (Khan et al., 2022a). In addition, inequality in healthcare services and/or limited access to alternative treatment, for example, for patients residing in rural areas or distant areas, halts the achievement of national TB-related targets (Khan et al., 2022a). With regard to healthcare professionals, Pakistan’s Ministry of Finance in Economic Survey 2020–2021 indicated that a single physician caters to the needs of hundreds of patients requiring treatment (Ministry of Finance, 2021). It was also reported that the mean consultation time (1.2 min–2.2 min) and the dispensing time (8.7 s–38 s) in the public sector facilities of Pakistan were suboptimal (Atif et al., 2016b; Atif et al., 2016c). Most of the pharmacists (20 per tertiary care hospital and 1–2 per secondary care hospital) performed their role in medicine management but did not provide any direct patient care.

Given the aforementioned facts, including increase in TB burden (World Health Organization, 2021), shortage of TB management staff and poor patient adherence, integration of pharmacists in TB management seems very much value-added (Kulkarni et al., 2013; Atif et al., 2016d; Khan et al., 2022a). Unfortunately, the importance of pharmacists in direct patient care is not recognized as an important and integral part of the Pakistani healthcare system (Atif et al., 2017a), regardless of the fact that the Drug Regulatory Authority of Pakistan (DRAP) Act 2012 acknowledges the advanced patient-oriented roles of pharmacists. Given that, research on this aspect was required to guide policy and practice reforms. Since physicians are key players in Pakistan’s healthcare system, their perspective on the patient-oriented roles of pharmacists was important. Moreover, it was equally important to know whether pharmacists were ready to take on these advanced roles. Being the ultimate beneficiary of any healthcare system, the perspective of patients was also required in this regard. Therefore, this study was conducted to gain insight into the perceptions of physicians, pharmacists, and patients about the role of pharmacists in TB management in Pakistan.

## Methods

### Study setting

The study was conducted at the Chest Disease Unit (CDU) of the Bahawal Victoria Hospital (BVH), Punjab, Pakistan. This tertiary care hospital has more than 1,600 beds and delivers services throughout all medical and surgical specialties. A monthly average of 90,000 patients is served by about 350 physicians, 20 pharmacists, 400 nurses, and 3,000 paramedics (Atif et al., 2017b). The CDU (60 bedded facility) is one of the major departments of BVH. 6–8 TB medical consultants, 3–5 medical officers, 6–8 postgraduate residents (serving inpatients), 6–8 house officers, one pharmacist, and 57 nurses serve TB patients and other patients with chest-related diseases. Currently, along with a permanent hospital-based pharmacist, a specialist pharmacist is appointed by the provincial TB control program, essentially supervising MDR-TB cases. The Global Fund and the National Tuberculosis Control Program of Pakistan (NTP) fund the TB sub-division of the CDU (Atif et al., 2016d).

### Study design

A qualitative study design was employed, where face-to-face interviews were conducted with healthcare professionals (i.e., physicians and pharmacists) and patients using a semi-structured interview schema. The inclusion criteria for the respondents are provided in Table 1. Sample size was determined by applying the saturation point criteria (Morse, 2000).

**TABLE 1:** Inclusion criteria.

Inclusion criteria
Physicians working in the chest disease unit for at least three months
Pharmacists currently working in the chest disease unit for at least three months or who had worked in the chest disease unit for at least one year
Patients aged ≥ 18 years of age suffering from pulmonary tuberculosis for at least three months

### Interview schema development

The interview schema was developed through formulating questions that answer the research problem and address gaps in the literature (Atif et al., 2016a; Atif et al., 2018; Khan et al., 2019; Atif et al., 2020a; Atif et al., 2020b; Atif et al., 2021). The patient and health professional interview schemas had six and seven questions (with additional sub-questions), respectively. The interview schema included questions about the importance of pharmacists in TB management, the roles that pharmacists currently play, and what roles are expected of them in the future. In addition, the barriers to and facilitators of integrating pharmacists into the TB management system and the perceived benefits of doing so were also inquired about. Before conducting the interviews, schema was administered to one participant from each cohort to ensure its uniformity and face validity. These pilot interviews were not included in the final analysis. To clarify the role of the pharmacist with the patients, Box 1 was used in the interview process (see Box 1).

BOX 1 **Role of the pharmacist explained to the patients**

• Provide medicine-related information
• Counsel the patient about medicine use
• Answer the queries of patients related to their medicines
• Assess the patient’s medication for appropriateness and safety
• Observe the effects of medicines in patients
• Early detection and resolution of adverse drug events in patients
• Design a therapeutic regimen tailored to an individual patient’s particular needs
• Promote adherence to medication

### Data collection

The study was conducted in three stages. In the first stage, physicians (registered with the Pakistan Medical and Dental Council) were interviewed to explore their perspectives regarding the roles of pharmacists in the TB management process. In stages 2 and 3, patients and pharmacists (registered with the Pakistan Pharmacy Council) were interviewed, respectively. Data were collected between 1st February and 31st May 2017. A mix of convenient and snowball sampling was used to engage healthcare professionals. Most of the healthcare professionals working in the CDU were difficult to reach because they were preoccupied with their routine clinical duties. Therefore, a convenient sampling method was deemed most appropriate, and interviews were conducted with individuals who were easiest to find and who consented to participate. After the initial stage of the convenient sampling method, a snowball sampling strategy was adopted, in which existing study subjects made recommendations and helped in the recruitment of future study subjects from among their colleagues and acquaintances. Patients were recruited using a convenient sampling method because they were reluctant to participate in the study and were exhausted due to long waiting hours.

### Data analysis

The data were analyzed using an inductive thematic analysis approach (Boyatzis, 1998). A verbatim translation of all the interviews was undertaken. The audio recordings of the interviews were listened to several times, and the written transcripts were also read several times inorder to immerse oneself in the data and develop a rich and deep understanding of it. After considerable discussion, meaningful data were extracted from each case, and particular codes were assigned to them. The coded data were read again and again and carefully analyzed to reduce and organize the same information and subsequently draw categories and themes (Starks and Brown Trinidad, 2007) Cross-checking was undertaken at each step of the analysis to ensure credibility and enhance the trustworthiness of the data (Lincoln and Guba, 1985).

### Ethics approval and consent to participate

Ethical approval was obtained from the Pharmacy Research Ethics Committee (PREC) at the Islamia University Bahawalpur (Reference: 42–2016/PREC). Prior to conducting interviews, the purpose of the study was explained to all participants (patients and healthcare professionals). Signed, written, informed consent was obtained from all study participants. The identity of the respondents (names and personal identifiers) was protected by assigning them a participant number.

## Results

A total of 30 healthcare professionals and 17 patients were initially approached, while 27 (90% response rate) healthcare professionals and 15 (88% response rate) patients consented to participate. Three healthcare professionals declined to participate in the study due to their busy work schedules (two) and lack of interest in the study (one). After reaching saturation point (14th interview for physicians, the 8th interview for pharmacists, and 11th interview for patients), one additional interview with each cohort of participants was conducted to confirm saturation in the emerged data. Among physicians, 10 were males and 5 were females, and among pharmacists, 4 were males and 5 were females. There were 10 male patients and 2 female patients. The average interview duration of physicians, pharmacists, and patients was 29.9 min (SD = 5.1), 28.4 min (SD = 5.3), and 22.2 min (SD = 2.7), respectively. The age range of the respondents was 2152 years. The characteristics of the respondents are provided in Table 2.

**TABLE 2:** Characteristics of the respondents.

Healthcare professionals				Patients			
Respondent	Gender	Specialization/qualification	Interview duration*(minutes)	Respondent	Gender	Qualification	Interview duration*(minutes)
Physician 1	Male	Pulmonologist	31	Patient 1	Male	^¶^Secondary	22
Physician 2	Female	Pulmonologist	29	Patient 2	Male	Secondary	18
Physician 3	Male	Pulmonologist	44	Patient 3	Male	Secondary	24
Physician 4	Female	Pulmonologist	32	Patient 4	Male	Secondary	25
Physician 5	Male	Pulmonologist	35	Patient 5	Male	Secondary	22
Physician 6	Male	Pulmonologist	32	Patient 6	Male	Secondary	25
Physician 7	Male	Pulmonologist	27	Patient 7	Male	Secondary	19
Physician 8	Female	Pulmonologist	29	Patient 8	Male	Secondary	20
Physician 9	Female	Pulmonologist	26	Patient 9	Female	^§^Primary	19
Physician 10	Male	Pulmonologist	32	Patient 10	Male	Secondary	22
Physician 11	Male	Graduate	24	Patient 11	Female	Primary	24
Physician 12	Male	Pulmonologist	23	Patient 12	Male	Secondary	26
Physician 13	Male	Pulmonologist	29				
Physician 14	Male	Pulmonologist	27				
Physician 15	Female	Pulmonologist	29				
Pharmacist 1	Male	Graduate	24				
Pharmacist 2	Female	Post graduate	26				
Pharmacist 3	Male	Post graduate	35				
Pharmacist 4	Male	Graduate	23				
Pharmacist 5	Female	Post graduate	28				
Pharmacist 6	Female	Post graduate	38				
Pharmacist 7	Female	Graduate	23				
Pharmacist 8	Female	Graduate	31				
Pharmacist 9	Male	Graduate	28				

*Rounded, ^§^primary (≤ 5 years of education), ^¶^secondary (6–13 years of education), tertiary (≥ 14 years of education).

### Thematic analysis

The data analysis process yielded seven themes and 19 categories representing the perspectives of study participants about pharmacists and their potential roles, rationale for integrating pharmacists in TB management, together with barriers and facilitators to efficient integration. For clarification, it is stated that patient-related findings under themes 1 and 3 depict their views before providing the information mentioned in Box 1 (potential role of pharmacists in TB management), while their views enclosed in themes 4 and 5 belong to a post-information inquiry. The emergent themes, categories, and supporting quotations are outlined in Table 3.

**TABLE 3:** Themes, categories, and supporting quotations from healthcare professionals and patients about the role of the pharmacist in TB management.

Categories	Supporting quotations
Theme 1: pharmacists: who are they?	
A qualified medicine expert	Physician 4: “A qualified person having a 5 years degree of Doctor of Pharmacy, who is an expert in medicines, who has a sound knowledge of the pharmacokinetics and pharmacodynamics of drugs, who is an expert in detecting and managing drug interactions and side effects, and who makes dose calculations according to the patient’s particulars. This is a pharmacist”
	Physician 14: “Someone who basically deals with drugs, their doses, their side effects, and their expiries. And who deals with a particular patient alongside with us. A person who has been dealing with drugs, their effects, side effects, and their dose, which will be selected according to the patient’s weight, is a pharmacist”
	Patient 6: “He is the one who gives the medicine, so I meet him every time I come. He counsels me on medicines, how to take it, when to take it. He assures me of the quantity being given to me for one month’s course. Then he allows me to leave the room”
Merely an unqualified dispenser	Patient 5: “We have heard and listened about doctor since we opened our eyes. We do not know about pharmacists. I have heard about people working in the medical store. They are not qualified enough. A doctor is well qualified and well educated”
	Patient 4: “No. What I know is that the person at the medical store who tells us about medicines and how much to take and how often…”
Theme 2: general responsibilities	
Inventory management	Physician 6: “A pharmacist is concerned with the availability of medicines in the department. He/she manages the stock, i.e., what medicines are being purchased, how to store those...”
	Pharmacist 9: “I am working as a hospital pharmacist in BVH. I am supervising the inventory of medicines and surgical items. I am supervising the dispensers; dispensing medicines to staff and to patients”
Dispensing of medicines	Physician 6: “A pharmacist is concerned with … dispensing of drugs, checking the expiry of the drugs”
	Pharmacist 9: “…I am supervising the dispensers, dispensing medicines to staff and to patients”
Theme 3: knowledge about the scope of practice of pharmacists in Pakistan	
Physicians’ perspective	Physician 8: “I do not know about laws. There were some amendments to the law. So we are not sure. According to my knowledge, this is a team work. Every job is not for the doctors and pharmacist is there to share our work. Together we can treat the patient in a better way, so it should be legal”
Pharmacists’ perspective	Pharmacist 4: “Legal is when you are permitted to do. A patient is coming to the hospital, and the most educated and qualified persons here are either doctors or us, the pharmacists. These duties are legal for us to perform. Whether we are permitted or not, it is a separate issue”
	Pharmacist 2: “This is our job, and by practicing all of these, the health system will get better. The patient’s care will improve. There will be fewer flaws and mistakes”
Theme 4: potential duties of pharmacists in the TB management	
Clinical duties	Physician 12: “Their basic work is inventory management … dose selection/adjustments, and inventory management … can be hired as treatment supporter … proper counseling … medication adherence assessment…”
	Pharmacist 9: “There should be a full-time, fully functional TB pharmacist whose responsibility should be to educate patients and their attendants about disease and therapy, to monitor the patients, to promote adherence to the treatment course, and to minimize the harms of therapy”
	Patient 12: “I want them to guide me. I shall take the medicine in a proper way and shall be cured soon with their guidance” (patient no. 12)
Prescribing	Pharmacist 8: “We are educated, we are experts in medicines, we have an authorized degree, we are supposed to assist doctors in finding better therapeutic alternatives, we are supposed to verify the doses, and then why should we not write a prescription. Diagnosis is not our field, it can never be, but once diagnosed by the doctors, we should be allowed to write a prescription while we are supposed to do all the other work”
Partial or differential diagnosis	Physician 4: “If a person comes to you with the complaint of pain in the epigastric region, there are numerous reasons for it. It can be simple heart burn or it can be angina or muscular pain and further sub categories of that. Now an untrained person cannot recognize it”
	Physician 5: “They do not need to do it. Diagnosis is not their job”
	Pharmacist 8: “Diagnosis is not our field, it can never be but once diagnosed by the doctors, we should be allowed to write a prescription when we are supposed to do all the other work”
	Patient 7: “Pharmacist’s job is confined to medicines and related aspects, not the diagnosis. Diagnosis is a specific work of the doctor”
Theme 5: rationale for integrating pharmacists into the TB care cascade	
Overburdened physicians	Physician 15: “We would like someone to share our burden or to help us. You know that diagnosis is our main task. So, sometimes the dosage and whether the drug is to be given through an IV route or an oral route, these things are confusing, and we have to search the internet, so, it will be a lot better when we have pharmacists, and they will just tell us”
	Physician 5: “Primarily, our burden will be shared. We will have more time for patients. We will be able to the study case thoroughly, diagnosis will be better”
Medication safety issues	Physician 14: “…if they will explain how to use the medicine and when to use, the side effects to the patients, compliance will be good…. Resistance will be overcome. Overall patient management will be better”
	Physician 14: “Pharmacists have a good command over pharmacology, if they will explain how to use the medicine and when to use, the side effects, to the patients, compliance will be good”
	Pharmacist 7: “We are unable to provide patient-centered TB care according to guidelines because we don’t have enough pharmacy workforce. Tackling medication safety issues is the responsibility of pharmacists in other countries. A single pharmacist cannot do this…I am overburdened. I have to look after two wards simultaneously, so the work is difficult to manage here … I will love to extend my duties if more staff is hired”
	Patient 12: “We do not know the appropriate way of taking medicine. That is why we often face the perilous effects of drugs, and the most dangerous is that the disease is not cured. If there will be a person to guide us, counsel us on the proper use of medicine and foods to take, we will get cured soon”
Poor patient-healthcare professional interaction and patient dissatisfaction	Patient 12: “Doctor did not attend me properly. He just saw the reports and went off. He did not discuss anything. I tried to talk to him about medicine, but he seemed angry. So I sat back. He wrote the prescription, and that’s all. He did not say a word or listen to me … there should be someone who can respond to us … like when I visit a medical store, the dispenser guides me about medicine and answers my questions, but here no one listens”
Theme 6: barriers to integrating pharmacists in the TB management	
Limited interest of regulatory authorities and policy-makers	Physician 15: “I think that is the government policies that matter here. I think the government is not creating the jobs because if vacancies are available obviously they will fill them. Other than that, hospital management does not realize their need and importance in the system, but I think it is policy related because there is a need of pharmacist”
	Pharmacist 2: “Main problem. Major issue. There are no proper policies for pharmacists. The rules are not being followed. The place, the seats we deserve are not being created. There is a need, there is demand for pharmacists, but it is not being fulfilled”
Lack of training	Physician 14: “Training can be an issue. Basically training at clinical side. The pharmacist who is working here, she is not having any clinical training. The duties that you have discussed earlier require clinical training”
	Pharmacist 4: “We are given training of only 6 weeks (internship) after studies, and that too does not involve any clinical training. So yes! We lack training”
Questionable competency	Physician 2: “I am not sure when to refer a patient to the pharmacist. Pharmacists themselves do not know what they are here for! This is a policy-related problem. Standard operating procedures for pharmacists are not clearly defined. They do not know whether they are here as hospital pharmacists or clinical pharmacists”
	Physician 1: “Honestly speaking, Yes! I do not consider them competent enough”
Lack of acceptability and awareness among community =	Physician 6: “It can be a problem. Our patients are not well educated. They are unaware of the importance of the pharmacist. From their view point, only the person who is diagnosing and prescribing is a doctor. Everyone else is just a supporter”
	Pharmacist 8: “Our community is mostly illiterate, they do not know who is a doctor and who is a pharmacist. Anyone giving them medicines is a doctor to them. They do not go and ask for your degree. They are concerned with the therapy and being cured”
Theme 7: facilitators to the integration of pharmacists in the TB management	
Appropriate training	Physician 8: “Yes, there should be some training and workshops for them. This is very important. The world is totally different in books and wards. In wards, there must be a supervisor for the pharmacist who can tell them how they can manage the disease”
	Pharmacist 9: “We have good knowledge of pharmacology; we also study diseases so we can better guide doctors on drugs and their actions and reactions on the patients. As far as training is concerned, our training should begin at undergraduate level and it should be a compulsory part of our course work. Training is required on the clinical side with proper supervision”
Improved awareness among community	Physician 15: “First of all create awareness among people. If the community do not know the basic infrastructure of the healthcare system like they does not know that what are the duties of nurses, doctors, and pharmacists”
Active participation of pharmacists in ward rounds	Pharmacist 1: “Pharmacists should work in wards, perform patient counseling, check drug interactions, and help counter medication/prescription errors. The patient will benefit from getting optimal treatment, and we shall gain experience”

#### Theme 1: Pharmacists: Who are they?

Most of the physicians (13 out of 15) described pharmacists as experts in medicines, having sound knowledge of the pharmacokinetics and pharmacodynamics of drugs. However, very few patients (3 out of 12) were aware of pharmacists and their duties. Most of the patients (9 out of 12) were completely unaware of the pharmacist and their role and believed that the pharmacist was merely a medication dispenser who was employed in medical stores and provided medicines to patients according to the physician’s prescription (Table 3).

#### Theme 2: General responsibilities

Most of the physicians (12 out of 15) and pharmacists (8 out of 9) stated that pharmacists were responsible for keeping an up-to-date record of the inventory items, ensuring the availability of medicines, maintaining inventory, and dispensing medicines (Table 3).

#### Theme 3: Knowledge about the scope of practice of pharmacists in Pakistan

Almost all physicians (13 out of 15) were unaware of the legal scope of pharmacy practice in Pakistan and whether responsibilities such as dose selection, clinical monitoring of the patients, patient counseling, etc., could be performed by pharmacists. However, they believed that these should come under their legal scope. On the other hand, pharmacists (9 out of 9) were quite certain that clinical activities, such as those outlined previously, were legislated for and expected to be undertaken by pharmacists (Table 3).

#### Theme 4: Potential duties of pharmacists in the TB management

When asked about the potential duties of pharmacists (after explaining the role of pharmacists to patients as described in Box 1), a number of physicians (13 out of 15), pharmacists (9 out of 9), and patients (7 out of 12) endorsed monitoring, counseling, brand selection, dose adjustment, inventory management, dispensing, and polypharmacy assessment as their potential duties in TB management. Almost all participants [physicians (14 out of 15), pharmacists (7 out of 9), and patients (11 out of 12)] agreed that pharmacists should not be involved in partial or differential diagnosis as it was not their field of work (Table 3).

#### Theme 5: Rationale for integrating pharmacists in the TB care cascade

The physicians (13 out of 15) said that they were overburdened and wanted to share their duties with pharmacists and nurses, preferably pharmacists. They elaborated that sharing responsibilities with pharmacists will help them have more time for other core clinical responsibilities. They also opined that patient counseling by pharmacists will facilitate improved treatment adherence and subsequently improve patient outcomes. Besides, pharmacists (8 out of 9) advocated that medication safety issues, for example, inappropriate use, medication errors, adverse drug events, etc., demand integration of the pharmacy workforce. However, they lamented that there was a shortage of pharmacist in TB management and a single pharmacists was unable to extend his/her duties due to excessive workloads. Harmoniously, patients (9 out of 12) accepted that pharmacists can better guide them about the proper use of medicines, thus reducing medication errors and improving their health status. In addition, patient satisfaction and effective consultation called for pharmacist integration into TB management, as patients (7 out of 12) were found complaining about the physicians’ behavior, lack of attention, and poor consultation style (Table 3).

#### Theme 6: Barriers to integrating pharmacists in the TB management

The major barrier to integrating pharmacists in the TB management system was the lack of interest of regulatory authorities and policymakers. Both the cohorts, i.e., physicians (15 out of 15) and pharmacists (8 out of 9), shared the same views on this matter. Physicians (14 out of 15) and pharmacists (8 out of 9) further expressed that though pharmacists hold relevant academic degrees (Doctor of Pharmacy), they still lack basic clinical training and experience, which influence their competency. Physicians (12 out of 15) were unclear about the competency and scope of pharmacy practice activities and did not know when it was appropriate to refer the patient to a pharmacist. One of the major barriers indicated by physicians (10 out of 15) was negative feedback from the community about the involvement of pharmacists in medicine-related decisions. A few pharmacists (3 out of 9) agreed with the physicians that patients were not familiar with their professional role or their services (Table 3).

#### Theme 7: Facilitators to the integration of pharmacists in the TB management

Nearly all physicians (14 out of 15) and pharmacists (9 out of 9) recommended that there should be arrangements for the clinical training of pharmacists, preferably at the undergraduate level. Most of the physicians (13 out of 15) and pharmacists (7 out of 9) were of the view that the community should be made aware of the role of pharmacists in TB management and the importance of engaging them in clinical activities. In addition to this, pharmacists (8 out of 9) suggested that they should join physicians on ward rounds to evaluate patients’ medication charts and assist them in therapeutic decision-making processes (Table 3).

**FIGURE 1:**
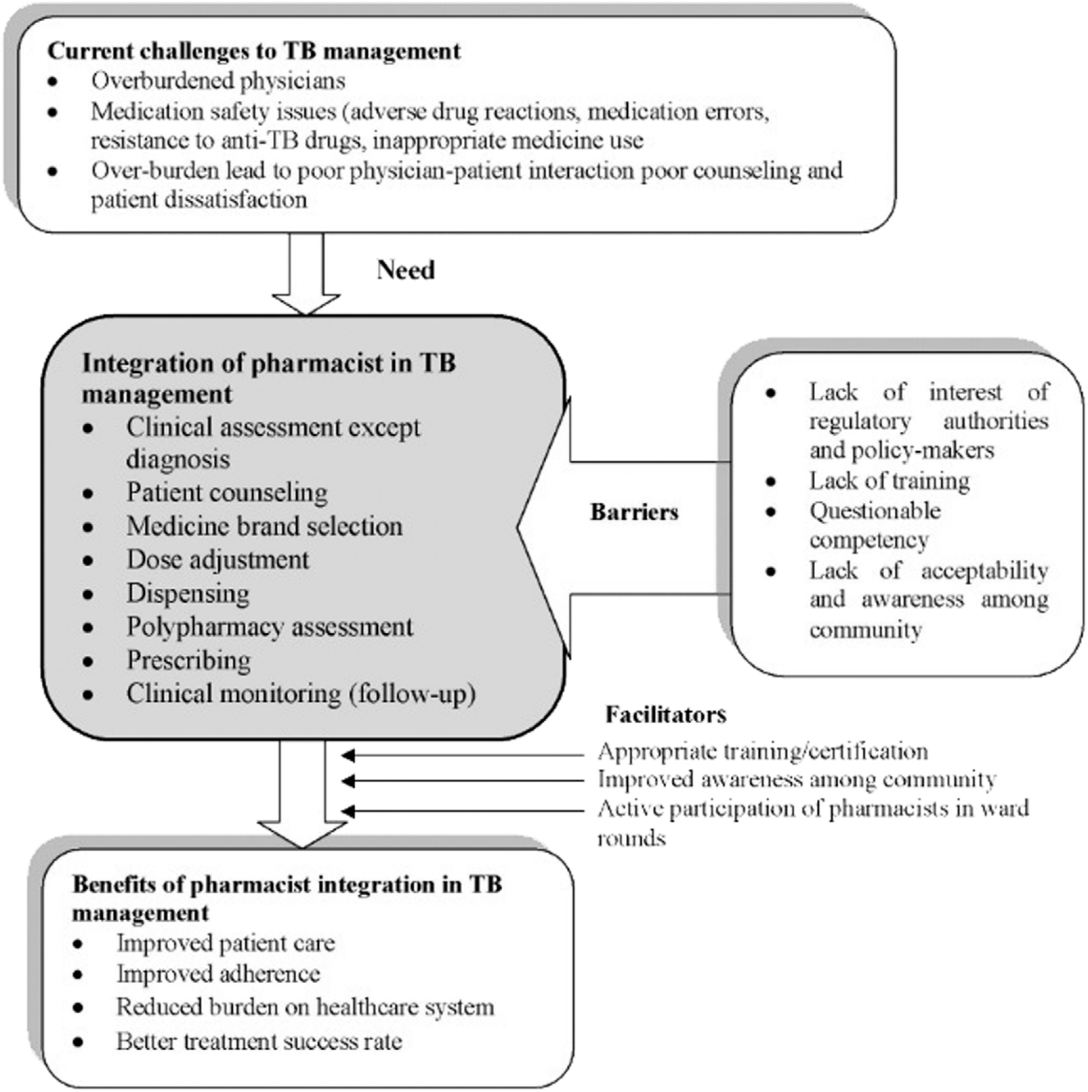
Summary of findings.

Figure 1 summarizes the need, barriers, facilitators, and benefits of integrating pharmacists in TB management.

## Discussion

It is widely acknowledged that integrating pharmacists in TB management improves therapeutic outcomes and patient safety and lessens the workload on infectious disease specialists (Atif et al., 2016b; Atif et al., 2016c). However, in Pakistan, the role of the pharmacist in the management of TB is restricted to the dispensing of medications only. Given the status quo, this study was conducted to understand the views of physicians, pharmacists, and patients with regard to integrating pharmacist roles in TB management, the potential duties pharmacists can perform, and the barriers linked to the integration of pharmacists in TB management. Analysis of the data yielded eight themes and 22 categories. The first six themes represented what is happening in the current scenario, whereas the latter two were related to future perspectives.

### Implications for the literature

In this study, physicians considered pharmacists to be “medicines expert” with the professional holding a responsibility of maintaining medicine inventories. Multiple studies (Azhar et al., 2010; Li et al., 2014; Sabry and Farid, 2014) demonstrated that, though physicians considered pharmacists as medicine experts, they were uncomfortable with their involvement in direct patient care. In the context of a developing country, this may be due to the fact that pharmacists were mostly restricted to medicine supply and distribution and were not involved in direct patient care (Adnan et al., 2014). Moreover, the current study revealed that most of the patients (when asked prior to explaining the potential role of pharmacists) were not aware of the existence of pharmacists and their potential role in TB management. The ignorant perception of patients may be attributed to a lack of education, poor health literacy, and limited access to pharmacy services. It is important to note that most of the TB patients in Pakistan have a low educational background and belong to rural areas (Atif et al., 2016a; Atif et al., 2018; Khan et al., 2019; Atif et al., 2020a; Atif et al., 2020b; Atif et al., 2021; Khan et al., 2022a). The same was reported in other studies (Ried et al., 1999; Wilbur et al., 2010; Adnan et al., 2014), where patients did not know the role of pharmacists, particularly in the provision of pharmaceutical care.

The healthcare professionals in the current study suggested that a pharmacist may provide patient-specific services such as assessing and promoting patients’ medication adherence and counseling the patients about the appropriate use of medicines and the importance of completing therapy. In addition, they also advocated that pharmacists could help physicians in monitoring the patients assessing the prescriptions for appropriateness and polypharmacy. Likewise, several studies (Clark et al., 2007; Mkele, 2010; Khan et al., 2022b) highlighted these roles of pharmacists in controlling and managing TB disease. Interestingly, in the current study, most of the pharmacists agreed that they were able to prescribe TB medicines to the patients. However, the legislations of pharmacy practice in Pakistan did not allow pharmacists to prescribe medicine for any disease. To date, Pharm-D graduates in Pakistan are not offered advanced training in pharmacotherapy and residency training to become independent prescribers. Nevertheless, in developed countries such as the United Kingdom, the United States, and Canada, where clinical pharmacy specialty courses are offered, pharmacists are allowed to prescribe (Tonna et al., 2007; Cooper et al., 2008; Stewart et al., 2009) after certification and necessary training, and patients consider pharmacists as competent and easily accessible healthcare professionals (Cooper et al., 2008).

The involvement of pharmacists in TB diagnosis was negatively viewed by almost all respondents to the current study. Though pharmacists in our study were not entrusted with the responsibility of diagnosis, a study (Glaze and Rowe, 2015) showed that involvement of pharmacists in this domain could help in the early detection of TB, thus preventing delays in treatment. A study conducted in New Mexico (Jakeman et al., 2015) found that the initial detection of presumptive TB patients by pharmacists resulted in valuable public health benefits. In countries with a high TB burden where healthcare professionals (especially physicians) are extremely overburdened, pharmacists can triage presumptive TB patients and refer them to physicians or the TB laboratory based on their needs. The same activities could also be performed by pharmacists at community pharmacies, which are often the first point of contact and consultation for presumptive cases with early symptoms of TB (i.e., cough, low grade fever, loss of weight, night sweats, etc.).

Nearly all respondents in this study demonstrated a positive response towards pharmacists’ authority to select any brand of prescribed medicines, and studies conducted in other countries showed almost similar findings (Hassali et al., 2009; Chong et al., 2010). Contrary to this, a study conducted in Jamaica (Gossell-Williams, 2007) found the opposite opinion of physicians in this regard. Jamaican physicians were not in favor of pharmacists undertaking substitution due to assorted reasons such as therapeutic failures, the occurrence of adverse drug reactions, etc. In the developing countries, for example Pakistan, pharmaceutical companies invest a lot on physicians to convince them to prescribe their brand. Understandably, in this case, physicians would not allow other health professionals to select a specific brand (Atif et al., 2019; Atif et al., 2020c). Interestingly, physicians in our study agreed to offer brand selection (for TB medicines) responsibility to pharmacists. This might be due to the fact that anti-TB medicines were available free of cost to the patient at public sector hospitals in Pakistan. Also, the physicians had to prescribe what was available in stock and they were not at the liberty to undertake selection of alternate brands available in market. The current study also revealed that the physicians did not agree that pharmacists could perform therapeutic substitution. This might be due to either the physician’s lack of trust in the pharmacist’s ability to select the appropriate medicine or they did not want pharmacists to interfere with their prescription-writing processes.

The majority of the stakeholders in this study were of the opinion that integration of pharmacists in TB management was direly needed in view of overburdened physicians, as excessive workload leads to sub-standard patient care. They raised concerns that poor counseling results in medication safety issues, such occurrence of ADEs associated with anti-TB drugs, medication errors, and the development of antibiotic resistance due to poor adherence to treatment, etc. Besides, the integration of pharmacists in TB management was also considered important in light of the meager patient-physician interaction and consequential patient dissatisfaction. Similar findings were also reported in previous studies (Clark et al., 2007; Mitrzyk, 2008; Mkele, 2010). Evidently, pharmacists are in an ideal position to educate TB patients about the disease and its management and subsequently help in reducing the occurrence of ADE associated with anti-TB drugs and improving compliance to TB treatment (Clark et al., 2007; Mitrzyk, 2008; Mkele, 2010; Atif et al., 2016d). Moreover, pharmacists in Pakistan could offer extended pharmacy services (Abrogoua et al., 2016; Atif and Malik, 2020; Atif et al., 2020d) at TB clinics, which may include pharmaceutical care, counseling, optimal use, pharmacoeconomics, and drug use evaluation. These roles are in line with the Drug Regulatory Authority of Pakistan (DRAP) Act no. XXI of 2012 (51).

With regard to barriers associated with the integration of pharmacists in TB management, physicians and pharmacists highlighted lack of interest by regulatory authorities, inadequate training, lack of acceptance, and questionable competency as major obstacles that hindered the integration of pharmacists in TB management. In addition, pharmacists also reckoned that a limited recruitment-driven shortage of pharmacists in the TB management unit imposed a burden on employed pharmacists, thereby making them unable to perform their duties efficiently. Similar barriers were reported in another study, which described how the limited availability of pharmacists makes it difficult for pharmacists to perform clinical roles in collaboration with physicians (Doucette et al., 2005).

### Implications for policy and practice

As with most exploratory studies, there are implications for policy and practice that come out of this work. First, Pakistani health policymakers need to be aware that physicians are significantly overburdened. Second, physicians see pharmacists as the “medicine experts,” yet are unaware of the legislation surrounding what is within the scope of a practicing pharmacist. There is a need for campaigns that aware physicians about the potential clinical roles of pharmacists. Third, various certification and residency programs should be available for pharmacists who want to improve their clinical skills. Finally, patients are unaware of the potential role of the pharmacist in their disease management. This requires education campaigns to change their perception.

### Limitations

There are some limitations to this study. First, the majority of the study patients were illiterate and from poor socioeconomic backgrounds. The perception of educated patients could have been different. Second, the study participants were only recruited from the Bahawalpur region; therefore, the results could not be generalized for the whole country. However, all TB management units in the country work under the National Tuberculosis Program and follow the same TB care cascade, in terms of staff availability, patient care services, medication, etc. Therefore, we believe that our findings can be generalized throughout the country. Third, the perspectives of nurses, policymakers, and NTP managers were not explored, which is recommended for future studies.

## Conclusion

Physicians considered pharmacists qualified healthcare professionals; whereas patients (pre-information inquiry) considered them merely dispensers. With regard to the potential duties of pharmacists, physicians, pharmacists, and patients [upon post-role-explanation (Box 1) inquiry] they endorsed monitoring, counseling, medicine brand selection, dose adjustment, inventory management, dispensing, and polypharmacy assessment as their potential roles. In view of all stakeholders, the rationale for integrating pharmacists in TB management included overburdened physicians, sub-standard patient care, medication safety issues, and patient dissatisfaction. The healthcare professionals highlighted that the major barriers to integrating pharmacists within the TB management system were limited interest of regulatory authorities and policymakers, followed by inadequate training and experience driven questionable competency of pharmacists. Moreover, awareness campaigns to sensitize healthcare professionals and the community about the legal role of pharmacists in patient-oriented services were deemed mandatory by the study participants.

## Data Availability

The original contributions presented in the study are included in the article/supplementary material, further inquiries can be directed to the corresponding author.
